# Draft genome sequence of *Streptomyces* sp. strain M41 isolated from soil in a conserved region of Sipadan Island, Sabah, Malaysia

**DOI:** 10.1128/mra.01309-24

**Published:** 2025-05-20

**Authors:** Mardani Abdul Halim, Nur Ariffah Waly, Gerald Jetony, Ken Kartina Khamis, Colin Robinson, Nurul Akmar Hussin, Clemente Michael Wong Vui Ling, Sazmal Effendi Arshad, Zarina Amin

**Affiliations:** 1 Biotechnology Research Institute, Universiti Malaysia Sabah 60606, Kota Kinabalu, Sabah, Malaysia; 2 Sabah Biodiversity Centre Natural Resources Office, Chief Minister’s Department, Kota Kinabalu, Sabah, Malaysia; 3 School of Bioscience, University of Kent 2240, Canterbury, United Kingdom; 4 Institute for Tropical Biology and Conservation, Universiti Malaysia Sabah 60606, Kota Kinabalu, Sabah, Malaysia; 5 Faculty of Science and Natural Resources, Universiti Malaysia Sabah 60606, Kota Kinabalu, Sabah, Malaysia; University of Strathclyde, Glasgow, United Kingdom

**Keywords:** streptomyces, genome, Sipadan Island

## Abstract

We present a draft genome of *Streptomyces* sp. isolated from soil in a conserved region of Sipadan Island, Sabah, Malaysia, and sequenced using the Illumina NovaSeq 6000. The draft genome consists of 70 scaffolds with a total length of 8,039,509 bp and a GC content of 70.9%. It contains 7,139 putative genes, including genes predicted to encode secondary metabolite biosynthesis.

## ANNOUNCEMENT

Streptomyces species are well known for their antimicrobial production and the synthesis of secondary metabolites due to specific environmental adaptation mechanisms (1, 2). *Streptomyces* sp. strain M41 was isolated from Sipadan Island, located in the Semporna District of Sabah, Malaysia, at latitude 4°7'0.33" N and longitude 118°37'40.972" E, using Actinomycete Isolation Agar (HiMedia, India). Briefly, 1 g of soil samples was weighed into a tube, and 9 mL of sterile distilled water was added to the tube to create a 10-fold dilution. The dilutions were spread on Actinomycete Isolation Agar without filtration and incubated at 25°C for 3 days under aerobic conditions. A single colony was picked from the growth and subsequently grown in actinomycete broth at 25°C for 3 days under aerobic conditions with continuous shaking prior to genomic DNA extraction. Genomic DNA was extracted using the Qiagen Genomic DNA Buffer Set Kit (Qiagen, Germany) following the manufacturer’s protocol. Identification was performed using 16S rRNA universal primers 27F 5′-AGAGTTTGATCMTGGCTCAG-3′ and 1492R 5′-TACGGYTACCTTGTTACGACTT-3′, and sequencing was done using the Sanger sequencing method. The output FASTA file was analyzed using BLAST (3) version 2.13.0 (https://blast.ncbi.nlm.nih.gov/Blast.cgi) against the core nucleotide database (core nt). Our analysis showed that *Streptomyces* sp. strain M41 was closely related to *Streptomyces* sp. strain G2R-M4-3-4 with 99.87% identity to the GenBank accession number PQ119703.1. For whole-genome sequencing, genomic DNA was enzymatically sheared into fragments of approximately 300 bp using the Illumina DNA Prep kit (Illumina, USA), followed by library preparation according to the manufacturer’s protocol. The library was then sequenced using the Illumina NovaSeq 6000, a 150 bp paired-end run with approximately 100× depth coverage, generating approximately 13.2 million reads. Unless otherwise noted, default parameters were used for all downstream bioinformatics analyses. The raw reads were adapter-trimmed using Trimmomatic v0.38 (4). The cleaned reads were assembled and scaffolded using SPAdes v3.15.3 (5). Subsequently, the assembled genome was annotated using the NCBI Prokaryotic Genome Annotation Pipeline v6.8 (6), with the option “minimum contig size (--mincontiglen)” set to 500 and an e-value cut-off of 1e-06. To screen for antibiotic-resistant genes, ABRIcate v1.0.1 was used (7). Genome-wide identification of secondary metabolite biosynthesis gene clusters was performed using antiSMASH v6.1.1 (8). The draft genome consisted of 70 scaffolds with a total size of 8,039,509 bp, an N50 of 183,107, and 70.9% GC. From the annotation, 6,928 coding sequence (CDS) were identified as protein-coding genes. ABRIcate analysis predicted two genes that might function in synthesizing antibiotics, namely oleandomycin and erythromycin. Genome-wide screening predicted 16 locations of possible secondary metabolite biosynthesis gene clusters, namely nonribosomal peptide synthetases (NRPS), terpene, RiPP-like lantipeptide, melanin, siderophore, ectoine, T3PKS, and T2PKS. The details of this analysis are summarized in Table 1.

**TABLE 1:** Predicted secondary metabolites gene cluster in *Streptomyces* sp. strain M41

Type	From	To	Most similar known cluster		Similarity (%)
NRPS	103,345	147,724	Diisonitrile antibiotic SF2768	NRP	66
Terpene	477,699	504,565	Hopene	Terpene	92
Terpene, butyrolactone	84,658	106,817	γ-Butyrolactone	Other	100
NRPS	1	37,426	Coelichelin	NRP	100
NRPS	85,747	147,159	Paenibactin	NRP	83
RiPP-like, lanthipeptide-class-iii	143,112	171,117	Informatipeptin	RiPP: lanthipeptide	100
Melanin	14,811	25,305	Melanin	Other	100
Siderophore	115,793	127,565	Desferrioxamin B/desferrioxamine E	Other	83
Terpene	114,686	140,092	Carotenoid	Terpene	63
NRPS, T2PKS, other, oligosaccharide	1	95,345	Komodoquinone B	Polyketide: type II+saccharide: hybrid/tailoring	100
Terpene	19,595	40,518	Albaflavenone	Terpene	100
Ectoine	13,619	24,023	Ectoine	Other	100
T3PKS	3,799	44,986	Germicidin	Other	100
Melanin, terpene	51,094	72,798	Melanin	Other	57
T2PKS	2,935	63,190	Spore pigment	Polyketide	75
Terpene	2,140	23,159	Neocarazostatin A	Other	100

## Data Availability

The draft genome was deposited in GenBank under the accession number JBIMKE000000000. Project data are available under BioProject PRJNA988168, with BioSample accession number SAMN36003836 and SRA accession number SRR25182456.
